# The role of RICTOR downstream of receptor tyrosine kinase in cancers

**DOI:** 10.1186/s12943-018-0794-0

**Published:** 2018-02-19

**Authors:** Ahlem Jebali, Nicolas Dumaz

**Affiliations:** 1 INSERM, U976, Centre de Recherche sur la Peau, Hôpital Saint Louis, F-75010, 1 avenue Claude Vellefaux, 75475 Paris cedex 10, Paris, France; 2 Univ Paris Diderot, Sorbonne Paris Cité, UMR 976, F-75010, Paris, France

**Keywords:** EGFR, HER2, mTOR, mTOR inhibitors, mTORC2, PDGFR, PI3K, RICTOR, RTK, VEGFR

## Abstract

The importance of the network defined by phosphatidylinositol-3-kinase (PI3K), AKT and mammalian target of rapamycin (mTOR) downstream of Receptor Tyrosine Kinase (RTK) has been known for many years but the central role of RICTOR (rapamycin-insensitive companion of mTOR) in this pathway is only starting to emerge. RICTOR is critical for mTORC2 (the mammalian target of rapamycin complex 2) kinase activity and as such plays a key role downstream of RTK. Alterations of RICTOR have been identified in a number of cancer cell types and its involvement in tumorigenesis has begun to be unraveled recently. Here, we summarize new research into the biology of RICTOR signaling in cancers focusing on tumors with altered RTK. We show that, as a key signaling node and critical effector of RTKs, RICTOR is becoming a valuable therapeutic target in cancer with altered RTK.

## Background

**Fig. 1:**
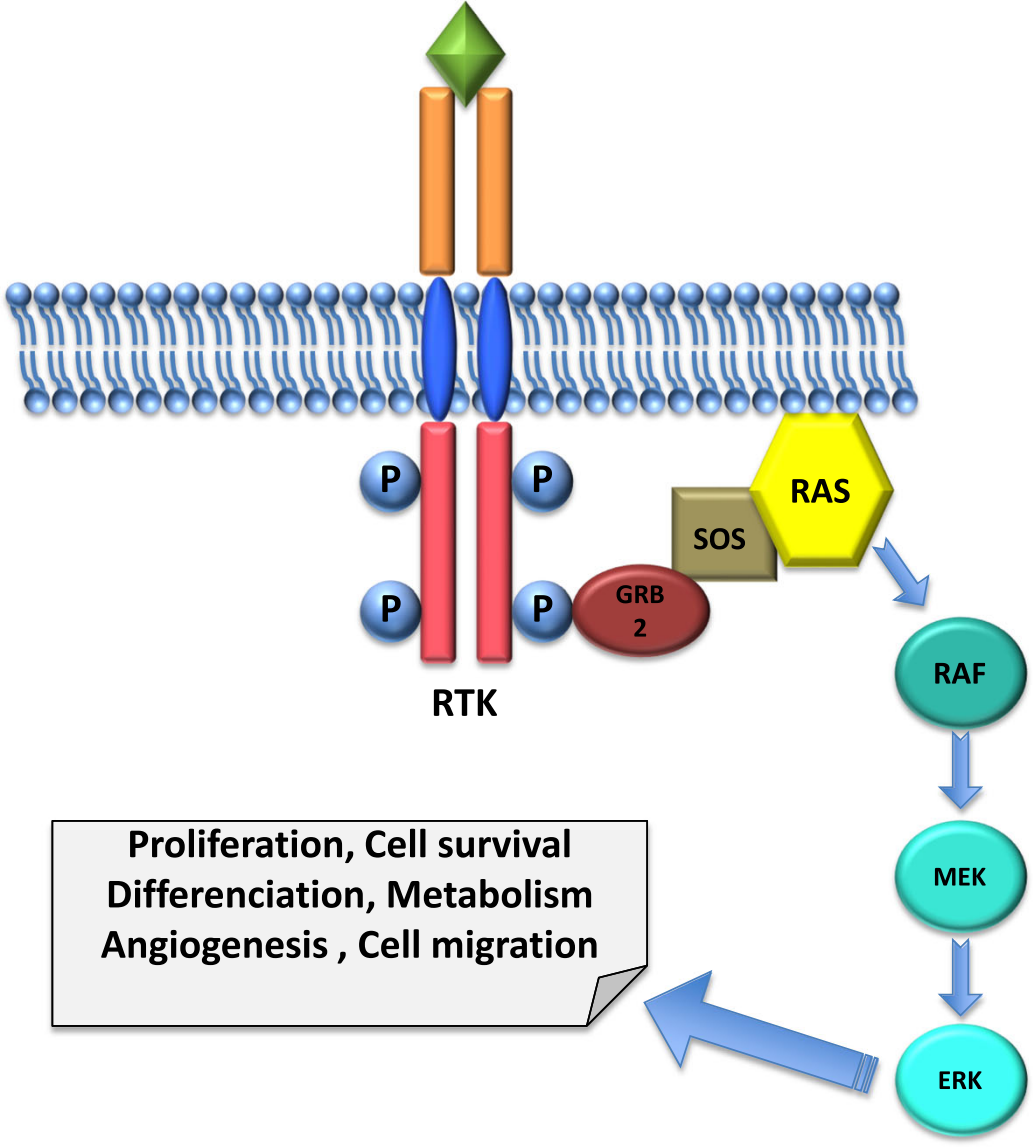
Schematic representation of a Receptor Tyrosine Kinase and the downstream MAPK pathway. The RTK is composed of a ligand-binding region in the extracellular domain, a transmembrane helix and a cytoplasmic region, which contains a tyrosine kinase domain. Its activation is due to a ligand-induced dimerization that results in receptor auto-phosphorylation of specific tyrosine residues in its intracellular domain. The GRB2 adaptor protein binds to the phosphorylated RTK and to the nucleotide exchange factor SOS that acts as a positive regulator of RAS allowing its interaction with the serine/threonine kinases of the RAF family, which activates MEK, which in turn activates ERK. ERK has many substrates, which control proliferation, differentiation, survival and migration

Receptor Tyrosine Kinases (RTKs) are a family of transmembrane receptors that mediate key signaling pathways in response to growth factors, cytokines, hormones, and other extracellular signaling molecules. RTKs drive a wide variety of essential processes such as cell proliferation, cell migration, differentiation and survival [1]. The RTK family includes, among others, epidermal growth factor receptors (EGFR), fibroblast growth factor receptors (FGFRs), insulin and insulin-like growth factor receptors (IR and IGFR), platelet-derived growth factor receptors (PDGFRs), vascular endothelial growth factor receptors (VEGFRs), hepatocyte growth factor receptors (HGFRs), and proto-oncogene c-KIT [2]. These receptors share a similar molecular architecture, with a ligand-binding region in the extracellular domain, a transmembrane helix, and a cytoplasmic region, which contains a tyrosine kinase domain [3]. Their activation is due to a ligand-induced dimerization that results in receptor auto-phosphorylation of specific tyrosine residues in its intracellular domain. These phosphorylation events create docking sites for Src homology 2 (SH2) domain-containing proteins, which in turn control various intracellular signaling pathways such as mitogen-activated protein kinases (MAPK), phosphatidylinositol 3-kinase (PI3K), phospholipase C-γ (PLCγ) and JAK/STAT [4] (Fig. 1).

Genetic changes that alter the activity, abundance, cellular distribution, or regulation of RTKs are observed in a wide variety of malignancies [5]. Gene mutations affecting EGFR members have been associated with several cancers. In breast cancer, overexpression of HER2 (Human Epidermal Growth Factor Receptor 2) is found in approximately 10–30% of patients [6]. Mutations affecting EGFR gene result in its overexpression in 30–50% of glioblastoma [7, 8], 25–82% in colorectal cancer [9] and 5–20% in non-small-cell lung cancer [10]. Mutations in the PDGFRα gene have been found in 5% of gastrointestinal stromal cancer (GIST) and amplifications of PDGFRα were reported in 5–10% of glioblastoma multiforme, in oligodendrocytoma, esophageal squamous cell carcinoma and artery intimal sarcomas [4]. Mutations in KIT are mostly found in leukemia, gastrointestinal stromal tumors (GIST), testicular germ cell tumor (TGCT) and melanoma [11]. These mutations affecting RTKs result in increased cell proliferation, survival, invasion and metastasis by activating downstream pathways such as the MAPK pathway and the PI3K pathway.

The MAPK pathway is one of the most deregulated signaling cascades in human cancer [12]. RTKs transmit signals to the MAPK pathway through the small GTPases of the RAS family. The GRB2 adaptor protein binds to the phosphorylated RTK through its SH2 domain and to the nucleotide exchange factor Son of Sevenless (SOS) by its SH3 domains. SOS acts as a positive regulator of RAS by promoting the exchange of nucleotide guanosine diphosphate (GDP) to nucleotide guanosine triphosphate (GTP). This exchange activates RAS, allowing its interaction with a number of effectors, in particular the serine/threonine kinases of the RAF family, which activate MAP kinase kinases (MEK), which in turn activate the MAP kinases (ERK). ERK has many substrates, which control proliferation, differentiation, survival and migration [13] (Fig. 1).

**Fig. 2:**
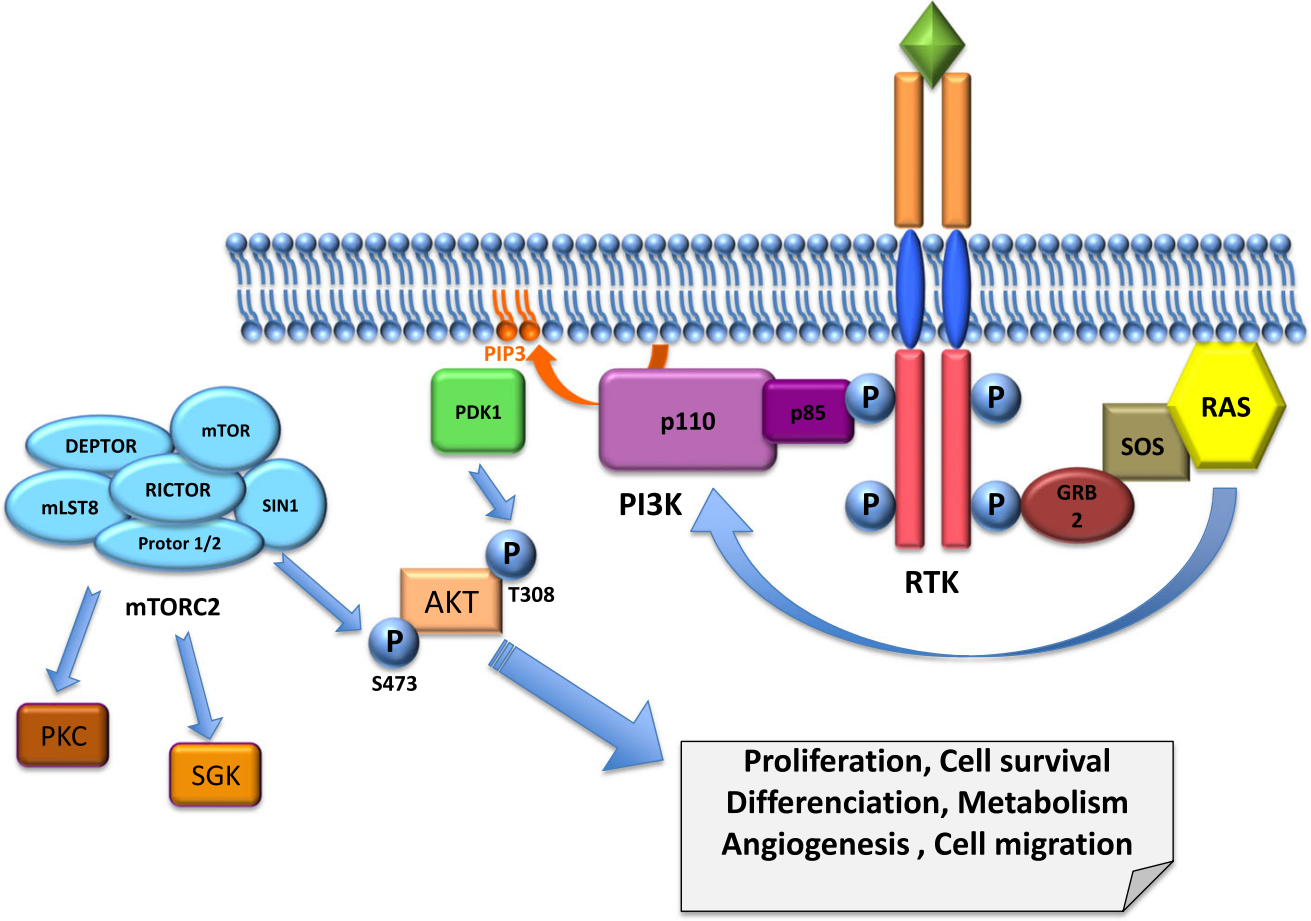
Schematic representation of an RTK and the downstream PI3K/AKT pathway. PI3K is activated downstream of RTKs by two mechanisms. First, a phosphorylated tyrosine residue on the receptor serves as a docking site for the p85 regulatory subunit of PI3K recruiting the catalytic subunit of PI3K, p110, to the plasma membrane. Second, activated RAS downstream of the RTK induces the membrane translocation and activation of the p110 subunit of PI3K. Activated PI3K converts PIP2 into PIP3, which is a docking site for PDK1 and AKT. AKT is then phosphorylated on Thr308 by PDK1 and on Ser473 by the mTOR kinase from the mTOR complex 2 (mTORC2). mTORC2 is defined by its scaffold protein RICTOR and promotes the stability and activation of AKT, SGK and PKC. AKT activates downstream signals involved in cell proliferation, differentiation, survival and migration

The PI3K pathway defined by PI3K, AKT and mammalian Target of Rapamycin (mTOR) controls most hallmarks of cancer, including proliferation, survival and motility, and contributes to cancer-promoting aspects of the tumor environment, such as angiogenesis [14]. It is activated downstream of RTKs by two mechanisms. First, a phosphorylated tyrosine residue on the receptor serves as a docking site for the p85 regulatory subunit of PI3K recruiting the catalytic subunit of PI3K, p110, to the plasma membrane. Second, activated RAS downstream of the RTK induces the membrane translocation and activation of the p110 subunit of PI3K [15]. Activated PI3K converts phosphatidylinositol 4,5 phosphate (PIP2) into phosphatidylinositol 3, 4, 5 phosphate (PIP3), which is a docking site for the pleckstrin homology (PH) domain of phosphoinositol-dependent kinase-1 (PDK1) and AKT. AKT is then phosphorylated on threonine 308 (Thr308) by PDK1 and on serine 473 (Ser473) by the mTOR kinase from the mTOR complex 2 (mTORC2) [13] (Fig. 2). mTOR kinase functions in association with different sets of proteins to form two distinct complexes, mTORC1 (mTOR complex 1) and mTORC2, which are large complexes with multiple protein components. Both complexes share the mTOR kinase, mLST8 (also known as GbL), DEPTOR, and the Tti1/Tel2 complex. mTORC1 also contains Regulatory-Associated Protein of Mammalian Target of Rapamycin (RAPTOR) and PRAS40, whereas mTORC2 contains Rapamycin-Insensitive Companion of mTOR (RICTOR), mSIN1, and Protor1/2 (Fig. 2). Compared to mTORC1, mTORC2 is insensitive to Rapamycin, due to its scaffold protein RICTOR. The majority of studies have focused on mTORC1; therefore, the regulations and functions of mTORC2 and the specific mechanism of RICTOR’s regulation of mTORC2 and other functions are less well-understood [16]. mTORC2 is the central component in the PI3K-AKT pathway, phosphorylating AKT at Ser473, causing its activation [17–19]. Other substrates of mTORC2 are AGC kinases, SGK and PKC, which have multiple functions in controlling cell survival, metabolic regulation, and cytoskeletal organization [20, 21].

RICTOR is a key component of mTORC2 and is required for mTORC2 function, shown by significant inhibition of the activation of AKT by RICTOR knockdown [19, 22]. Therefore, as a critical regulator of the PI3K/AKT pathway, RICTOR plays an important role in tumors driven by RTK alterations. In addition, the *RICTOR* gene has recently been shown to be amplified in cancer, highlighting its role in cancer development and its potential as a therapeutic target.

A detailed understanding of the molecular mechanism that underlies RTK-induced tumorigenesis is essential for the development of effective therapeutic strategies for this subset of tumors. This review highlights the important role played by RICTOR downstream of RTK in tumoral cells and the potential of targeted inhibition of RICTOR/mTORC2 in the treatment of tumors with alterations of RTK signaling.

### RICTOR amplification and overexpression in cancer

**Fig. 3:**
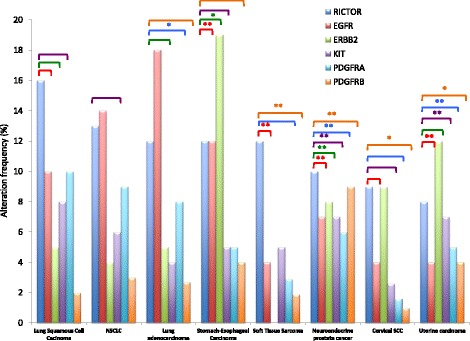
Frequencies of alterations of RICTOR, EGFR, ERBB2, KIT, PDGFRA and PDGFRB in several tumor types. Brackets indicate a tendency towards co-occurence of RICTOR and RTK alterations. When the co-occurence is statistically significant, it is indicated with * (*P* < 0,05) or ** (*P* < 0,01). Data are derived from the publicly available TCGA datasets and obtained through the cBioPortal for Cancer Genomics. NSCLC: Non Small Cell Lung Cancer. SCC: Squamous Cell Carcinoma

Several studies have demonstrated an amplification of the *RICTOR* gene or an overexpression of its protein in different cancer types. Among RICTOR-amplified samples the most common tumor-types are neuroendocrine prostate cancer (18%) and lung squamous cell carcinoma (16%), followed by sarcoma (12%) and esophagus and stomach cancer (10%). Interestingly RTK alterations have also been identified in these tumors and analysis of the available databases through the cBioPortal for Cancer Genomics shows a tendency for co-occurrence of RICTOR and RTK alterations in these tumors (see TCGA Data Portal; [23, 24] (Fig. 3).

*RICTOR* was identified as the most frequently amplified gene observed (~ 14% patients) in a cohort of metastatic small cell lung cancer (SCLC), where *RICTOR* copy number variation correlated with RICTOR protein expression in SCLC cells. The overall survival in SCLC patients with *RICTOR* amplification was significantly decreased [25]. Moreover, analysis of the Cancer Genome Atlas (TCGA) database for *RICTOR* alteration demonstrated that *RICTOR* was amplified in around 13% (132/1016) of patients with lung cancers, including 10.3% in lung adenocarcinoma (53/515) and 15.8% (79/501) in squamous cell carcinoma [26]. Interestingly, in a subset of 85 cases with *RICTOR* amplification, 41% (35/85) presented at least one alteration in an RTK gene (EGFR, HGFR, FGFR, ALK, KIT etc.) [26]. A study of 640 patients with metastatic solid tumors (primarily gastrointestinal and lung cancers) confirms the amplification of *RICTOR* in lung cancer and demonstrated that *RICTOR* amplification was rare but recurrent in gastric cancer (GC). The prevalence of *RICTOR* amplification detected by NGS and confirmed by FISH, in patients with GC was 3.8% (6/160) [27]. Elevated RICTOR expression was also found in GC and directly correlated with tumor size, invasion of stomach wall, infiltration of lymph node and vessels, tumor stage and differentiation. These results suggest that RICTOR is associated with tumor progression and poor prognosis in patients with GC and might therefore be used as a novel biomarker for prognosis [28]. In a cohort of 201 cases of esophageal squamous cell carcinoma (ESCC) RICTOR expression was estimated by immunohistochemistry and associated with clinicopathological parameters. The percentage of RICTOR-positive expression was 70.6% (142/201), which correlated positively with ESCC patients’ AJCC (American Joint Committee on Cancer) stage and was associated with a bad prognosis. Expression of RICTOR and AJCC staging III or IV were independent risk factors for ESCC [29]. A study of the *RICTOR* locus by CGH array in a series of 43 melanoma short-term cultures showed that *RICTOR* was amplified in 19 out of 43 melanoma cell lines (44%) and that amplification was independent of the BRAF and NRAS mutation status, the most frequent mutations in melanoma. Quantification of RICTOR mRNA in 22 melanoma short-term cultures confirmed that *RICTOR* locus amplification was associated with an increase in RICTOR mRNA level [30]. In breast cancers, RICTOR was enriched in HER2-amplified samples and correlated with increased phosphorylation of AKT at S473, consistent with a potential role for mTORC2 in HER2-amplified breast cancers. In invasive breast cancer specimens, RICTOR expression was upregulated significantly compared to non-malignant tissues [31]. Besides lung cancer, ESCC, melanoma, GC and breast cancer, RICTOR overexpression was also reported in glioblastoma [32, 33], hepatocellular carcinomas [34] and pancreatic ductal adenocarcinoma (PDAC) [35].

Because RICTOR plays a key role in mTORC2 formation and AKT activation, it may also play a key role in the tumorigenic potential of altered RTK. RICTOR deregulation could have important effects in tumor development either because it cooperates with altered RTKs to transform cells or as a critical regulator of a major pathway downstream of RTKs.

### RICTOR effects on cell proliferation, cell survival and angiogenesis

The correlation between RICTOR overexpression, tumor progression and poor survival in a variety of cancers suggests that RICTOR amplification plays a role on cell proliferation, cell survival or tumor microenvironment. We summarize below the recent research into the biology of RICTOR signaling in cancers in which RTK signaling plays a major role.

#### Breast cancers

The importance of the PI3K/AKT signaling is well documented in HER2-amplified breast cancer models and the role of RICTOR/mTORC2 is becoming increasingly recognized. Inhibition of mTORC1/2, by mTOR kinase inhibitors PP242 and OSI-027 or RICTOR knockdown, effectively suppressed phosphorylation of AKT (S473) and breast cancer cell proliferation and migration. It also promoted serum starvation- or cisplatin-induced apoptosis and prevented breast tumor growth in vivo in a xenograft model [36]. RICTOR/mTORC2 has also been found essential for the ability of HRG (EGF-like growth factor) to promote transformation of HRG-sensitive breast cancer cells. Disruption of the mTORC2 arm of the pathway via the knockdown of RICTOR, significantly attenuated the ability of HRG to promote HER2-dependent oncogenesis [37]. These results were confirmed in a HER2/Neu mouse model of breast cancer, where RICTOR ablation decreased AKT S473 phosphorylation, cellular proliferation and delayed tumor latency, burden and penetrance suggesting that RICTOR promotes the genesis of HER2-overexpressing tumors [31]. Therefore, HER2-amplified breast cancers use RICTOR/mTORC2 signaling to drive tumor formation, tumor cell survival and resistance to HER2-targeted therapy. mTORC2 inhibition may offer a promising therapeutic strategy to help eradicate HER2-amplified breast cancers, in particular in tumors which are resistant to HER2 targeted therapy or where AKT signaling is activated.

#### Lung cancers

RICTOR amplification has been reported in lung cancer and was associated with a decrease in overall survival. *RICTOR* copy number variation correlated with RICTOR protein expression in SCLC cells [25]. Its oncogenic roles were suggested by decreased lung cancer cell growth both in vitro and in vivo with RICTOR ablation, and the capacity of RICTOR to transform Ba/F3-cell [26]. SCLC cell lines with various levels of RICTOR copy number (CN) gain were used to analyze its downstream effects on cell growth and migration. The authors showed that SCLC cell lines with *RICTOR* CN gain migrated more rapidly compared to cells with no gain in *RICTOR* CN, associating *RICTOR* amplification with increased cell motility [25]. Lung cancer cells with RICTOR amplification showed increased sensitivity to mTORC1/2 inhibitors, whereas silencing RICTOR rendered *RICTOR*-amplified cells markedly more resistant to mTORC1/2 inhibitors, demonstrating that RICTOR was the target in those cells [25]. Interestingly in a cell line combining RICTOR and PDGFR amplification, *RICTOR* knockdown was associated with significantly reduced proliferation in vitro and in vivo, consistent with RICTOR’s role as an oncogenic driver downstream of PDGFR [26]. The subset of lung cancer patients with RICTOR amplification may benefit from drugs targeting mTORC1/2. Indeed, a patient with a lung adenocarcinoma presenting a *RICTOR* amplification demonstrated tumor stabilization for 18 months upon treatment with mTORC1/2 inhibitors [26].

#### Pancreatic cancers

The PI3K/mTOR pathway functions downstream of RAS, which is mutated in 90% of PDAC, and plays a key role in IR/IGFR signaling which is overexpressed in pancreatic cancer tissues. RICTOR/mTORC2 are increasingly recognized as important players in pancreatic cancer development. Expression of RICTOR in PDAC is associated with reduced survival in patients [35]. Knockdown of RICTOR by RNA interference in human pancreatic cancer cell lines has an inhibitory effect on tumor growth in vitro and in vivo [35]. Using a PDAC genetically engineered mouse model (GEMM), it was also shown that RICTOR deletion dramatically delayed tumor formation, whilst mice with median survival almost doubled in RICTOR-deleted mice compared with control mice [38]. The knockdown of RICTOR in two primary PanIN (pancreatic tumor precursor) cell lines established from mice with early PanIN, reduced proliferation in both cell lines and enhanced the expression of senescence-associated beta galactosidase [38]. Pharmacological inhibition of mTORC1/2 delayed tumor formation and prolonged survival in late-stage tumor. In conclusion, these results provide evidence for mTORC2/ RICTOR as an attractive novel target for treatment of human PDAC.

#### Colon cancers

Activation of the PI3K/AKT/mTOR signaling pathway is associated with growth and progression of colorectal cancer (CRC). More specifically, increased expression of RICTOR is associated with tumor progression and poor survival in CRC (32), and mTOR activity and complex distribution are independent prognostic factors in colorectal carcinoma [39]. Inhibition of mTORC1/2 signaling, using pharmalogical inhibitors or knockdown of mTORC1/RAPTOR and mTORC2/RICTOR, attenuated migration and invasion of CRC cells, induced a mesenchymal–epithelial transition and enhanced chemosensitivity of CRC cells to oxaliplatin [40]. Selective inhibitors of TORC1/2 caused growth suppression in CRC cells in vitro and in vivo and enhanced the anticancer activities of doxorubicin in colorectal xenograft mouse models [41]. In CRC cells, RICTOR expression is also regulated by the miR-424/503 cluster, which contributes to tumor progression. RICTOR is upregulated via the repression of the miR-424/503 cluster in colon cancer cell lines that harbor c-SRC upregulation. The re-expression of miR-424/503 caused downregulation of RICTOR, and decreased tumorigenicity and invasive activity of these cells. Furthermore, downregulation of miR-424/503 is associated with RICTOR upregulation in colon cancer tissues [42]. Finally, a relationship between autophagy and RTK activation through mTORC2 signaling was recently identified in CRC cells. c-MET has a tumor-promoting role in CRC and has been characterized as a resistance mechanism to EGFR-targeted therapy. Basal autophagy positively regulates c-MET activation via an mTORC2-mediated mechanism [43]. These findings provide the rationale for including mTORC1/2 inhibitors as part of the therapeutic regimen for CRC patients.

#### Glioblastoma

Amplification of the gene encoding the EGFR occurs commonly in glioblastoma (GBM), the most common malignant primary brain tumor of adults. EGFR overexpression leads to activation of downstream kinases, including the PI3K/AKT/mTOR pathway. mTORC2 is frequently activated in GBM and both EGFR and RICTOR are associated with increased proliferation, invasion, metastasis and poor prognosis. [33]. mTORC2 signaling promotes GBM growth and survival downstream of EGFR. mTORC2 activates NF-κB, which renders GBM cells and tumors resistant to chemotherapy in an AKT-independent manner. mTORC2 inhibition reverses chemotherapy resistance in vivo [33]. The co-silencing of EGFR and RICTOR in GBM cell lines resulted in reduced cell migration, and increased sensitivity to vincristine and temozolomide. While the silencing of EGFR or RICTOR alone had no significant effect on xenograft tumor growth in vivo, silencing of EGFR and RICTOR simultaneously resulted in a complete eradication of tumors suggesting that the combined silencing of EGFR and RICTOR should be an effective means of treating GBM [44]. Recently, a small molecule, which specifically blocks the interaction of RICTOR and mTOR, was developed as a potential inhibitor of mTORC2 activity in GBM. In vitro, it inhibited mTORC2 kinase activity at submicromolar concentrations, and, in cellular assays, specifically inhibited phosphorylation of mTORC2 substrates without affecting the phosphorylation status of the mTORC1 substrate. This inhibitor demonstrated significant inhibitory effects on cell growth, motility and invasiveness in GBM cell lines, and sensitivity correlated with relative RICTOR or SIN1 expression. In GBM xenograft studies, this small molecule demonstrated significant anti-tumor properties [45]. These results highlight the critical role of mTORC2 in the pathogenesis of GBM, including tumors with altered EGFR. These findings suggest that therapeutic strategies targeting mTORC2, alone or in combination with chemotherapy or EGFR inhibition, could be effective in the treatment of GBM.

#### Gastric cancers

An elevated RICTOR expression is associated with tumor progression and poor prognosis in patients with GC whereas no significant association is observed between mTORC1 activity and clinicopathological features or prognosis, suggesting that mTORC2 plays a more important role than mTORC1 in gastric tumor progression [28]. Stable sh-RNA mediated down-regulation of RICTOR, significantly inhibited GC cell proliferation, migration and invasion, and enhanced apoptosis [46]. Furthermore, *RICTOR* amplification defines a subset of advanced GC that displayed increased sensitivity to the dual mTORC1/2 inhibitor, AZD2014, and the dual PI3K/mTOR compound, BEZ235, whereas AKT inhibitor AZD5363 had lesser effects on *RICTOR*-amplified patient-derived cell growth. RICTOR knockdown was sufficient to abrogate the inhibitory effects of AZD2014 on cell growth, consistent with the functional importance of RICTOR amplification [27]. Together, these data support the oncogenicity of *RICTOR* amplification and provides the rationale for targeting both mTORC1 and mTORC2 as part of the therapeutic strategy for GC.

#### Tumor microenvironment

In addition to its direct effects on tumoral cells described above, RICTOR also plays a role in tumor progression by regulating the tumoral microenvironment either through angiogenesis or through remodeling of the stroma. In pancreatic tumors, it was shown that RICTOR blockage led to an inhibition of hypoxia-induced factor-1α (HIF-1α) expression and a significant reduction of its downstream target vascular-endothelial growth factor-A (VEGF-A), a critical cancer-promoting factor involved in the recruitment of stromal cells [35]. Similarly, in prostate cancer, it was demonstrated that miR-218 inhibited the tumor angiogenesis of prostate cancer cells in vitro and in vivo via the regulation of RICTOR expression. RICTOR knockdown phenocopied miR-218 overexpression in inhibiting prostate cancer angiogenesis. These findings revealed an important involvement of the RICTOR/VEGF axis in tumor progression via the mechanism of angiogenesis [47]. In melanoma, where *RICTOR* amplification and overexpression are frequent, down-regulation of RICTOR with shRNA severely impaired the formation of vasculogenic mimicry (VM) via the AKT-MMP-2/9 pathway. The pathological investigation showed that melanoma tissues overexpressing RICTOR are prone to form VM channels, and this formation was accompanied by AKT membrane translocation and an increase in MMP-2/9 secretion [48]. These results support the hypothesis that RICTOR regulates VM formation.

Taken together, these studies attest that RICTOR amplification and overexpression play a role in tumor growth, at least in part via vascularization and remodeling of the tumoral stroma.

### RICTOR as a therapeutic target

The importance of the PI3K/AKT/mTOR pathway in cancer has been known for many years but the central role of RICTOR in this pathway is only starting to emerge. In many cancer types, it was shown that RICTOR overexpression in tumoral cells leads to an increase in cell proliferation and survival, and a decrease in cell apoptosis in cancer cells as well as a remodeling of the stroma, which all favor tumor development. Interestingly, overexpression of RICTOR was positively associated with tumor progression and poor survival in colorectal cancer [40], hepatocellular carcinoma [34], endometrial carcinoma [49], pituitary adenoma [50] and PDAC [35]. RICTOR is therefore becoming an important actor in cancer diagnosis, prognosis and treatment.

RICTOR is frequently overexpressed in tumoral cells, often due to gene amplification. Moreover, in absence of gene amplification, RICTOR overexpression can also be associated with the deregulation of miRNA expression in tumoral cells such as miR-218 in prostate and oral cancers, the miR-424/503 cluster in colon cancers, and miR-196b in melanoma and hepotocellular carcinoma [42, 47, 51, 52]. Besides gene amplification and miRNA, RICTOR overexpression can also be linked to transcription factors and epigenetic modifications. For instance, the transcription factor FoxO elevates the expression of RICTOR, leading to increased mTORC2 activity while inhibiting mTORC1, thereby activating AKT. FoxO may act as rheostat that maintains homeostatic balance between AKT and mTOR complexes activities [53, 54]. Also, the histone dimethyl transferase WHSC1 was recently shown to transcriptionally upregulate RICTOR expression to further enhance AKT activity to promote prostate cancer metastasis, highlighting the role of the AKT/WHSC1/RICTOR cascade in prostate cancer malignancy [55].

Although most reports demonstrate the important role of RICTOR via the RTK-PI3K/AKT pathway activation, mTORC2/RICTOR also exhibits AKT-independent activities, which could play a role in the oncogenic potential of RICTOR. It has been shown that the adaptor PRICKLE1 interacts with RICTOR, controls actin cytosqueleton organization and contributes to breast cancer cells dissemination [56]. Disruption of the PRICKLE1-RICTOR interaction resulted in a strong impairment of breast cancer cell dissemination in xenograft assays. It was also shown that upregulation of PRICKLE1 is associated with AKT signaling and poor prognosis in basal breast cancers [56]. In another study, it was shown that mTORC2 uses two coordinated pathways to drive breast cancer metastasis, one AKT-dependent and one AKT-independent, both of which converge on RAC1. AKT signaling activated RAC1 through the RAC-GEF TIAM1, while PKC signaling dampened expression of the endogenous RAC1 inhibitor, RHOGDI2 [57]. RICTOR has also been shown to be an important component of FBXW7 E3 ligase complex participating in the regulation of c-MYC and CYCLIN E protein ubiquitination and degradation, and also in RICTOR stability [58, 59]. Finally, RICTOR not only acts downstream of IGF-IR/InsR, but also seems to regulate activation of IGF-IR/InsR. A recent study showed that the mTORC2 complex has a dual specificity kinase activity and directly promoted IGF-IR/InsR activation [60]. The role of these AKT-independent activities of RICTOR/mTORC2 in cancer development is not completely clear yet and will need to be validated.

As a key signaling node and critical effector of RTKs, RICTOR/mTORC2 has become a valuable therapeutic target. The first generation of mTOR inhibitors (rapamycin and rapalogs; Table 1) only targeted mTORC1. Their use for the treatment of cancers has shown a limited response rate [61] in part due to a strong feedback loop between mTORC1 and AKT, which activated the latter. The second generation of ATP-competitive mTOR inhibitors that target both mTORC1 and mTORC2 (Table 2) has shown greater effectiveness than rapalogs for cancer treatment. However, the mTORC1 inhibition-induced negative feedback activation of PI3K/PDK1 and AKT (Thr308) may be sufficient to promote cell survival [62]. The recent studies demonstrating that mTORC2 activity is essential for the development of a number of cancers provide a rationale for developing inhibitors specifically targeting mTORC2, which do not perturb the mTORC1-dependent negative feedback loops and have a more acceptable therapeutic window. To date, mTORC2 specific inhibitors are not available and targeting RICTOR remains difficult due to its lack of enzymatic activity. However, RICTOR is a direct target of the ribosomal protein S6 kinase-1 (S6 K1) that phosphorylates it on Thr1135 and mediates 14–3-3 binding to RICTOR, inducing a conformational change that prevents mTORC2 from phosphorylating AKT (41). mTORC2 inhibition by RICTOR phosphorylation on Thr1135 could be used as a novel strategy for specifically inhibiting mTORC2. Furthermore, small molecules, which specifically block the interaction of RICTOR and mTOR, have been developed and could be used as specific inhibitors of RICTOR/mTORC2 and an alternative to mTORC1/2 inhibitors [45]. The role of RICTOR in RTK driven tumors has begun to be unraveled, and targeting RICTOR/mTORC2 could have therapeutic impact in these tumors. RICTOR/mTORC2 inhibition may therefore offer a promising therapeutic strategy to treat RTK-altered tumors, specifically those which are resistant to RTK targeted therapies.

**Table 1:** First generation of mTOR inhibitors

Name	Structure
Rapamycin	Macrocyclic lactone with two binding moietics
Temsirolimus (CCI-779)	Moietics modification: dihydroxylmethyl propionic acid ester
Everolimus (RAD001)	Moietics modification: Hydroxylethyl group
Ridaforolimus (AP23573)	Moietics modification: Dimethyl phosphate group

**Table 2:** Second generation of mTOR inhibitors

dual PI3K/mTOR inhibitors		Selective mTOR inhibitors	
Structure	Name	Structure	Name
Pyrimidine derivative	PI-103 group include: GDC-0980, GNE-493, GNE-477, PF-04691502	Pyrimidine derivative	PP242, PP30, INK128
Imidazoquinoline derivative	BEZ-235 group include: BGT226, GKS2126458	Morpholino-linked pyrimidine derivatives	WAY-600, WYE-687, WYE354, KU0063794, AZD8055
Quinoxaline derivative	XL765	Triazine derivative	OSI-027

### Conclusions and perspectives

As a key player in mTORC2 formation and AKT activation, RICTOR plays a significant role downstream of RTK. The importance of RICTOR downstream of RTK in cancer is highlighted by the fact that not only can alterations of RICTOR and RTK co-occur in some tumors, but also that RICTOR expression is essential to permit the oncogenic potential of RTKs such as HER2, PDGFR, or EGFR. Although significant progress has been made in developing small molecule inhibitors and monoclonal antibodies that target components of the RTK signaling pathways in cancer, an important obstacle remains in the capacity of cancer cells to adapt to these inhibitors by developing resistance. Inhibitors targeting RICTOR/mTORC2 may be valuable tools to treat RTK-altered tumors which are resistant to therapies targeting RTKs.

## Abbreviations

AJCC: American joint committee on cancer
CRC: Colorectal cancer
EGFR: Epidermal growth factor receptor
ESCC: Esophageal squamous cell carcinoma
FGFR: Fibroblast growth factor receptor
GBM: Glioblastoma
GC: Gastric cancer
GDP: Guanosine diphosphate
GIST: Gastrointestinal stromal cancer
GTP: Guanosine triphosphate
HER2: Human epidermal growth factor receptor 2
HGFR: Hepatocyte growth factor receptor
HIF-1α: Hypoxia-induced factor-1α
IGFR: Insulin-like growth factor receptor
IR: Insulin receptor
MAPK: Mitogen-activated protein kinases
mTOR: Mammalian target of rapamycin
mTORC1: Mammalian target of rapamycin complex 1
mTORC2: Mammalian target of rapamycin complex 2
PDAC: Pancreatic ductal adenocarcinoma
PDGFR: Platelet-derived growth factor receptor
PDK1: Phosphoinositol-dependent kinase-1
PH: Pleckstrin homology
PI3K: Phosphoinositide 3-kinase (mTOR)
PIP2: Phosphatidylinositol 4,5 phosphate
PIP3: Phosphatidylinositol 3,4,5 phosphate
PLCγ: Phospholipase C-γ
RAPTOR: Regulatory-associated protein of mammalian target of rapamycin
RICTOR: Rapamycin-insensitive companion of mTOR
SCLC: Small cell lung cancer
SH2: Src homology 2
SOS: Son of sevenless
TGCT: Testicular germ cell tumor
VEGF-A: Vascular-endothelial growth factor-
VEGFR: Vascular endothelial growth factor receptor
VM: Vasculogenic mimicry
